# Male-Dominated Migration and Massive Assimilation of Indigenous East Asians in the Formation of Muslim Hui People in Southwest China

**DOI:** 10.3389/fgene.2020.618614

**Published:** 2021-01-11

**Authors:** Qiyan Wang, Jing Zhao, Zheng Ren, Jin Sun, Guanglin He, Jianxin Guo, Hongling Zhang, Jingyan Ji, Yubo Liu, Meiqing Yang, Xiaomin Yang, Jinwen Chen, Kongyang Zhu, Rui Wang, Yingxiang Li, Gang Chen, Jiang Huang, Chuan-Chao Wang

**Affiliations:** ^1^Department of Forensic Medicine, Guizhou Medical University, Guiyang, China; ^2^Department of Anthropology and Ethnology, Institute of Anthropology, School of Life Sciences, Xiamen University, Xiamen, China; ^3^WeGene, Shenzhen, China

**Keywords:** genetic structure, population genetics, southwest China, population admixture, population history

## Abstract

The origin and diversification of Muslim Hui people in China via demic or simple cultural diffusion is a long-going debate. We here generated genome-wide data at nearly 700,000 single nucleotide polymorphisms (SNPs) from 45 Hui and 14 Han Chinese individuals collected from Guizhou province in southwest China. We applied principal component analysis (PCA), ADMIXTURE, *f*-statistics, *qpWave*, and *qpAdm* analysis to infer the population genetic structure and admixture history. Our results revealed the Guizhou Hui people have a limited amount of West Eurasian related ancestry at a proportion of 6%, but show massive genetic assimilation with indigenous southern Han Chinese and Tibetan or Tungusic/Mongolic related northern East Asians. We also detected a high frequency of North Asia or Central Asia related paternal Y-chromosome but not maternal mtDNA lineages in Guizhou Hui. Our observation supports the cultural diffusion has played a vital role in the formation of Hui people and the migration of Hui people to southwest China was probably a sex-biased male-driven process.

## Introduction

The Hui people are an East Asian ethnoreligious group distributing throughout China with a population of approximately 20 million predominantly composed of Chinese speaking practitioners of Islam (Mu, 1985). Although the majority of Hui people speak Han Chinese language nowadays, their culture and food habits have distinct differences with Han Chinese. The origin and diversification of Hui groups via demic diffusion involving the mass movement of people from West Eurasia and the Middle East or simple cultural diffusion with massive assimilation of indigenous East Asians is a long-going debate. The historical records suggest the origins of Hui people were mainly in two different periods: first, starting from the Tang dynasty (around seventh century AD), the Persians and Arabs came to the southeast coast of China for trade and then gradually mixed with local East Asian populations, especially with the Han Chinese. Second, the Central Asians, Persians and Arabs came to China following the Mongol invasions and conquests during the thirteenth and fourteenth centuries in the Yuan Dynasty (Bao, 1982; Du and Yip, 1993; Gladney, 1998). The immigration was suggested to be male-dominated involving a large number of soldiers, merchants, and political emissaries (Du and Yip, 1993).

Previous studies from the genetic perspective revealed that the origin of Hui people in China had involved massive assimilation of indigenous East Asians inferred from paternal Y-chromosomal single nucleotide polymorphism (SNP) and short tandem repeat (STR) analysis (Zhang et al., 2010; Lan et al., 2018; Wang et al., 2019; Xie et al., 2019). From the maternal mitochondrial DNA (mtDNA) side, Western Eurasian related lineages were found in Hui people of Xinjiang in northwest China, but only accounting for a low frequency at about 6.7% (Yao et al., 2004). The autosomal STR analysis on Hui people in Gansu province in northwest China showed genetic homogeneity of the Muslim populations and local East Asian populations, with no evidence of substantial gene flow from the Middle East or Europe into Hui people during their Islamization (Xie and Shan, 2002; Yao et al., 2016). Previous studies have shed some light but far from giving a comprehensive and conclusive understanding of the origin of Hui people due to the limited markers used. Besides, the majority of previous genetic studies on Hui people have focused on northern China, but the Hui people in southern China are seldom investigated.

Guizhou province in southwest China is a multi-ethnic province with abundant of genetic and cultural diversities. The Han Chinese have the largest proportion of the population in Guizhou, accounting for 62.2% of the total population of the province. The Muslim Hui is about 90,000 people mainly distributing in Weining County, accounting for 0.5% of the total population in Guizhou province. Weining County was the earliest place for the Hui people moving to Guizhou Province (Bao, 1982). According to historical records, the ancestors of the Hui people had already arrived in Guizhou in the Yuan Dynasty (Bao, 1982). The origin of the Chinese Hui is always controversial though considerable studies have been performed. For exploring the origin and genetic structure of Guizhou Hui, in this study, we generated genome-wide data including 699,537 paternal, maternal phylogenetic relevant SNPs and autosomal SNPs on Guizhou Hui samples using array genotyping. In addition, we also sampled Guizhou Han as a reference population to facilitate the analysis on the assimilation of indigenous ethnic groups in Guizhou Hui people. Here we aimed to explore the origin and genetic admixture of Guizhou Hui people and shed light on the understanding of early Chinese Hui migration patterns from a genetic perspective.

## Materials and Methods

**FIGURE 1:**
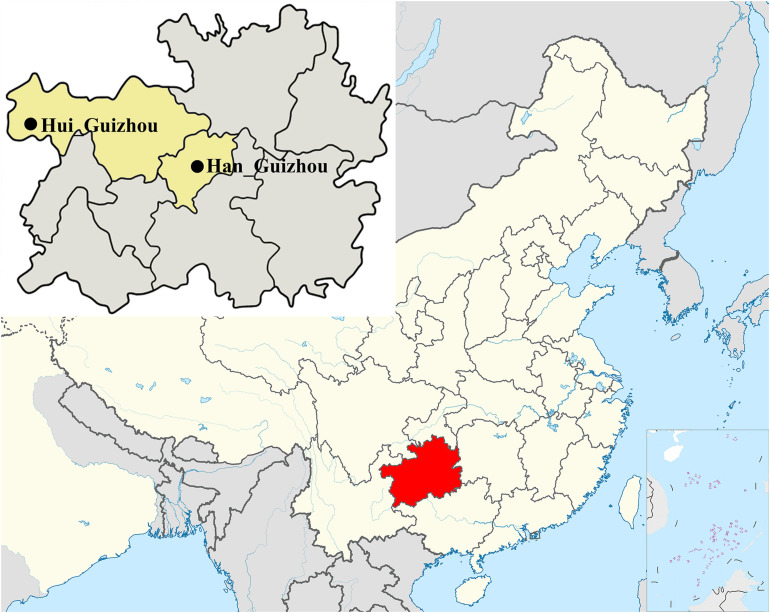
Geographic locations of sampling marked with black solid circles, including 45 Hui and 14 Han individuals in Guizhou province of southwest China. Detail information for each samples was listed in Supplementary Table S1.

We collected 59 saliva samples in Guizhou province with informed consent, including 45 Hui people from Weining County and 14 Han people from Guiyang city. We collected the samples following genetic population criteria. These samples enrolled in the present study were collected randomly from unrelated participants whose parents and grandparents are indigenous people and have the non-consanguineous marriage within the same ethnical group for at least three generations. We listed the detailed sample information in Supplementary Table S1 and the geographic locations of sampling in Figure 1. Of all samples, 23 were male and used for Y-haplogroup paternal analysis. All individuals were performed for maternal mtDNA analysis.

Genomic DNA of 59 samples were extracted using DP-318 Kit (Tiangen Biotechnology, Beijing). The DNA quality control was carried out at the experimental center of WeGene-Shenzhen. Genotyping was performed on the Illumina WeGene Arrays at the WeGene genotyping center, Shenzhen. We merged the data of Guizhou Hui and Han individuals with published populations from Human Origin Dataset (Patterson et al., 2012; Lazaridis et al., 2014), Simons Genome Diversity Project (SGDP) (Mallick et al., 2016), 1000 Genomes Project (1000 Genomes Project Consortium et al., 2015), and also ancient East Eurasian samples from 1240K capture dataset curated by David Reich lab^1^.

**FIGURE 2:**
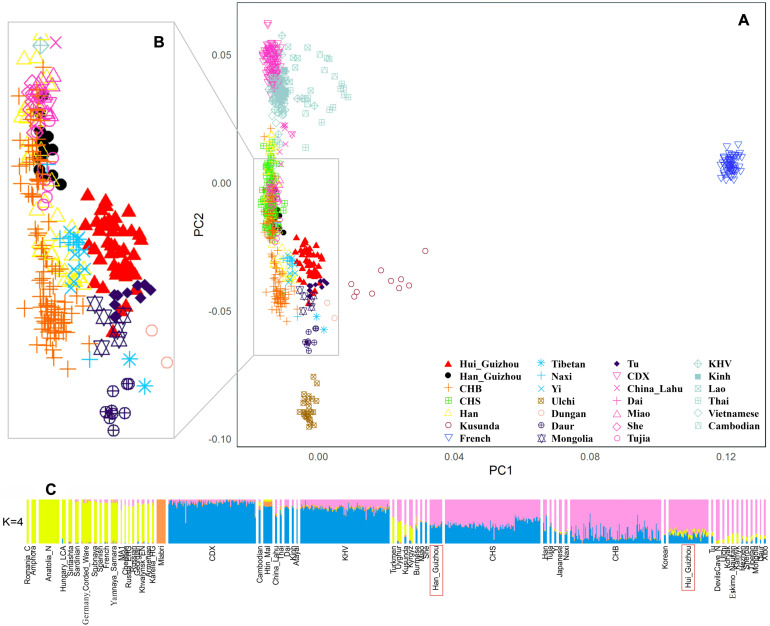
Genetic structure of analyzed populations in this study. **(A)** Principal component analysis of Hui_Guizhou and Han_Guizhou with other East Asian and French populations. **(B)** An enlarged view of the area marked by the gray wireframe in **(A)** with CHS (Southern China) removed. **(C)** ADMIXTURE model-based clustering analysis of Hui_Guizhou and Han_Guizhou groups with present and ancient worldwide populations. The results showed when four ancestral sources are predefined.

We carried out Principal Component Analysis (PCA) using smartpca, part of the EIGENSOFT package (Patterson et al., 2006). We used default parameters with the numoutlieriter: 0 settings and assessed statistical significance with a Tracy-Widom test by the twstats program of EIGENSOFT. We discussed and plotted principal components in what follows were highly statistically significant (*P* < 10^–12^). We carried out ADMIXTURE (Alexander et al., 2009) analysis by the following steps: we firstly pruned SNPs in strong linkage disequilibrium with each other using PLINK tools with the parameters “-indep-pairwise 200 25 0.4”; we then run ADMIXTURE with the K values (number of assumed ancestral components) ranging from 2 to 10 applying 100 bootstraps iterations with different random seeds. The best run was chosen according to the highest likelihood. An optimal K value was selected using 10-fold cross-validation plotted in Figure 2C.

We used ADMIXTOOLS (Patterson et al., 2012) to compute *f*-statistics and determined standard errors with a block jackknife and default parameters. We computed outgroup *f*_3_-statistics of the form *f*_3_ (Mbuti; X, Y), which measured the shared genetic drift between the two populations X and Y since their separation from an African outgroup Mbuti. We computed admixture *f*_3_-statistics of the form *f*_3_ (Target; Source 1, Source 2) to explore possible sources for Hui_Guizhou and Han_Guizhou people. We also calculated the *f*_4_-statistics of the form *f*_4_ (X, Y; Test, Outgroup) to show if population Test is symmetrically related to X and Y or shares an excess of alleles with either of the two. Moreover, we used *qpAdm* (Haak et al., 2015) as implemented in ADMIXTOOLS with the option “allsnps: YES” to test the number of sources of ancestry that is needed to estimate the admixture proportions of Guizhou Hui population with the proposed sources. The outgroups selected are differentially related to the ancestral sources of Guizhou Hui people.

The Y chromosomal haplogroups were assigned by identifying the most derived upstream allele and the most ancestral downstream allele in the phylogenetic tree in the ISOGG version 11.89^2^. The mtDNA haplogroup assignment was determined with mtDNA tree Build 16 (van Oven and Kayser, 2009)^3^.

## Results

### Genetic Structure of Hui and Han Ethnic Groups in Guizhou

We first carried out a PCA to obtain a qualitative picture of how Guizhou Hui and Han individuals related to previously published East Asian populations from Human Origin Dataset (Patterson et al., 2006; Lazaridis et al., 2014). We used the names “Hui_Guizhou” and “Han_Guizhou” to refer to samples from Hui and Han ethnic groups in Guizhou province, respectively. The result was shown in Figure 2A. To facilitate observation, we enlarged the area of interest (gray wireframe in Figure 2A) on the left (Figure 2B) by removing CHS (Han Chinese in southern China) to avoid visual clutter. Based on the first and second principal components, Guizhou Hui and Han individuals were divided into two distinct genetic clusters. In addition, we found that Hui_Guizhou people clustered closely with northern populations (such as Mongolic-speaking, Sino-Tibetan, Tu, and CHB (Han in Beijing, China) populations) rather than with southern populations. The genetic structure of Hui_Guizhou was not in accordance with their geographical distribution. Unlike Hui_Guizhou, Han_Guizhou overlapped with populations in southern China, such as CHS, She, Miao and Tujia.

The results of model-based ADMIXTURE clustering analysis (Alexander et al., 2009) were consistent with PCA showing that Hui_Guizhou population had a closer affinity on average to northern populations instead of southern groups (Figure 2C and Supplementary Figure S2). Figure 2C showed the ADMIXTURE results assuming *K* = 4 clusters (we selected this number because it was the most optimal K value using 10-fold cross-validation). The primary ancestry component assigned to the Hui_Guizhou population, shown in purple, also maximized in Neolithic DevilsCave hunter-gatherers (Siska et al., 2017; Sikora et al., 2019) and present-day Ulchi individuals in Russian Far East. ADMIXTURE also assigned to Hui_Guizhou samples an ancestry component of yellow color, which maximized West Eurasians but was absent in the southern Chinese populations. The ancestry composition of Hui_Guizhou shown in Figure 2C indicated that there were more northern related, followed by the southern related, and a few West Eurasian related ancestry components. Han_Guizhou people displayed an analogous genetic profile with southern Chinese populations, like CHS, Miao, She and Tujia in terms of admixture proportions related to southern and northern components.

### Population Continuity and Admixture in the Hui_Guizhou and Han_Guizhou

The outgroup *f*_3_-statistics (Raghavan et al., 2014) of the form *f*_3_ (Mbuti; X, Y) were consistent with the patterns observed in the above PCA and ADMIXTURE analysis, suggesting that Hui_Guizhou shared more genetic drift with Tu and Tibeto-Burman speaking populations (Supplementary Figure S1). However, Han_Guizhou showed close genetic proximity with southern Chinese populations, especially Hmong-Mien speaking populations, Tujia and CHS. In addition, we used negative Z-scores of admixture *f*_3_-statistics of the form *f*_3_ (Target; Source 1, Source 2) to explore possible sources for Hui_Guizhou and Han_Guizhou people in this study. The results in Supplementary Table S2 showed that the top possible related sources of Hui_Guizhou consisted of Han related especially Han_Guizhou in this study, Hmong-Mien speaking populations, and ancient and present-day West Eurasians. The top possible sources of the Han_Guizhou were Atayal, Dai and northern-related populations such as Tibetan, Ulchi and Hezhen groups. Inferred from the *f*_3_-statistics (Supplementary Table S2), although the genetic structure of Hui_Guizhou was closer to northern related populations, we still found the signal of possible genetic assimilation with indigenous southern related populations, like Han_Guizhou and Hmong-Mien speaking populations (Supplementary Figure S3).

To further explore the differentiation between the Hui_Guizhou and indigenous populations, we performed the *f*_4_ statistics (Patterson et al., 2012) in the form of *f*_4_ (Test, Mbuti; Hui_Guizhou, Han_Guizhou). Supplementary Table S3 showed that present-day and ancient Europeans shared more alleles with Hui_Guizhou people than with Han_Guizhou, suggesting West Eurasian gene flow into Hui_Guizhou. We confirmed the results by replacing Han_Guizhou with other Han Chinese groups in datasets (Supplementary Table S4). In addition, we continued to calculate *f*_4_ statistics in the form of *f*_4_ (Test, Mbuti; Hui_Guizhou, She) and *f*_4_ (Test, Mbuti; Hui_Guizhou, Miao), respectively (Supplementary Tables S5, S6). Similarly, East Asian populations shared more alleles with Miao and She groups than with Hui_Guizhou, while Hui_Guizhou people tended to be closer to Western Eurasian-related populations.

### The Ancestry Related Sources of Hui_Guizhou

**FIGURE 3:**
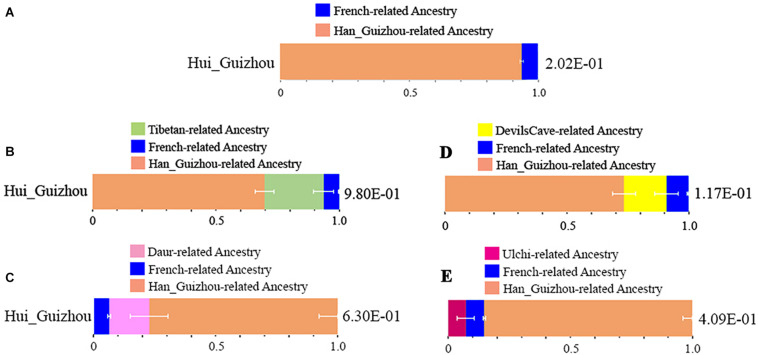
Modeling the ancestral source of Hui_Guizhou people via *qpWave* and *qpAdm.* **(A)** the proportions of French and Han_Guizhou related ancestry. **(B)** the proportions of Tibetan, French, and Ha_Guizhou related ancestry. **(C)** the proportions of Daur, French, and Han_Guizhou related ancestry. **(D)** the proportions of DevilsCave, French, and Han_Guizhou related accestry. **(E)** the proportions of Ulchi, French, and Han_Guizhou related ancestry. All the modeling results for the Hui_Guizhou cluster and related outgroups were showed in Supplementary Tables S7–S12.

We systematically explored diverse *qpAdm*-based admixture models for identifying plausible admixture sources for Hui_Guizhou people in this study. Firstly, we used Han_Guizhou and French as proxies for the East Asian and western related source populations in a two-way admixture. Hui_Guizhou individuals were estimated to have 6.2% French related and 93.8% Han_Guizhou related ancestry (Figure 3A and Supplementary Table S7). We then replaced Han_Guizhou with Han Chinese in published datasets to confirm the estimation (Supplementary Table S8). We observed a consistent result regardless of whether one of the proxies was Han Chinese or Han_Guizhou.

Furthermore, we were particularly interested in East Asian related sources in Hui_Guizhou people. We were wondering if we could distinguish the Han_Guizhou related local southern component and Tibetan/Tungusic related northern ancestry. We proposed a more complex three-way model using Han_Guizhou, Tibetan, and French as three sources (Supplementary Table S9 and Figure 3B). In the East Asian related sources, Han related ancestry had the largest proportion accounting for 69.6%, while Tibetan related ancestry also had a prominent proportion of 24%. We then replaced Tibetan with ancient DevilsCave (Supplementary Table S10 and Figure 3D) and present-day Ulchi (Supplementary Table S11 and Figure 3E) in the Russian Far East, as well as Daur (Supplementary Table S12 and Figure 3C) in northern China. The admixture proportion of Neolithic DevilsCave related nomadic ancestry was estimated to be 17.4% in Hui_Guizhou. The Daur related ancestry was also estimated at a similar proportion of around 16.3% in Hui_Guizhou. However, we observed a difference in the ancestry proportion of present-day Ulchi related sources in Hui_Guizhou, which was estimated to be 7.4%. These results above indicated the ancestors of Hui people in Guizhou had a large amount of Tibetan/Tungusic related northern ancestry before they migrated to southwest China.

### MtDNA and Y Chromosomal Haplogroup Analysis

**FIGURE 4:**
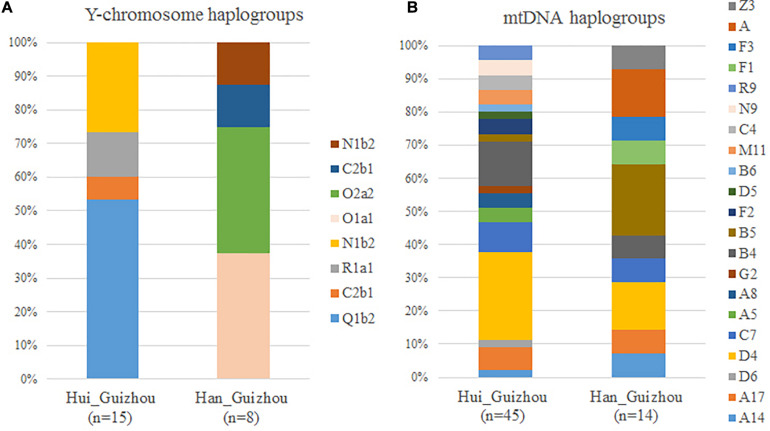
Comparison of Y-chromosome **(A)** and mitochondrial haplogroups **(B)** distribution in the Hui_Guizhou and Han_Guizhou lineages.

We showed the maternal mtDNA haplogroups of Hui_Guizhou and Han_Guizhou samples in Supplementary Table S13 and Figure 4B. Although we found a high diversity of the lineages in the maternal gene pool of Hui_Guizhou and Han_Guizhou, these mtDNA haplogroups detected were all frequent in East Asian populations. We observed a genetic North-South admixture pattern in the haplogroup frequency profile of mtDNA. In detail, haplogroup D4, D5 and D6 accounted for 31.11% (14/45) in Hui_Guizhou and 14.29% (2/14) in Han_Guizhou individuals. Previous studies suggested that these haplogroups were predominant in populations from northern China (Tanaka et al., 2004; Wen et al., 2004; Kong et al., 2011; Li et al., 2019). Haplogroup G2 and Z found in Hui_Guizhou and Han_Guizhou were also relatively abundant in northern China (Tanaka et al., 2004; Li et al., 2007). In addition, haplogroup B, F and R were mainly found in southwest Chinese and Southeast Asian populations (Tanaka et al., 2004; Li et al., 2007), contributed to 26.67% (12/45) of Hui_Guizhou and 42.86% (6/14) of Han_Guizhou people. The other lineages, like haplogroup A and N9, contributed to 17.78% (8/45) in Hui_Guizhou and 28.57% (4/12) in Han_Guizhou populations, which had a prevailing northern Asia dispersal (Tanaka et al., 2004; Li et al., 2019). Haplogroup C and its sublineages were considered to have expanded in Northeast Asia (Tanaka et al., 2004), accounting for 13.33% (6/45) of Hui_Guizhou and 7.14% (1/14) of Han_Guizhou people.

To trace the paternal history and genealogical ancestors of Hui and Han Chinese in Guizhou, we performed Y-chromosome haplogroups analysis. On the paternal side, a striking feature of the Y chromosomal profile of Hui_Guizhou was the high frequency of haplogroup Q1b2b1b2a-L330-F1893 (Supplementary Table S13 and Figure 4A), accounting for 53.33% (8/15) of the total Hui samples. Haplogroup Q1b2b1b2a-L330-F1893 was a subclade of Q-L330. Q-L330 was the major sub-lineage of the Q-M242 samples in populations of southern Siberia and the adjacent region of the Mongolia Plateau, like the Altaians, Tuvans, and Kets (Dulik et al., 2012; Karmin et al., 2015). Previous studies had revealed the haplogroup Q-M242 might have originated in southern Siberia and diffused gradually to other parts of Eurasia since the Paleolithic Age (Dulik et al., 2012; Balanovsky et al., 2017; Huang et al., 2018). The second frequent haplogroup was N1b2a2-M1811 accounting for 26.67% of Hui_Guizhou people. N1b2a2-M1811 was the sub-clades of N1b-F2930, which was prevalent in the present-day Sino-Tibetan populations (Ilumäe et al., 2016). R1a1a1b2-F992 and R1a1a1b1a2b3-FGC4499/Y2192 were subclades of haplogroup R1a1a-M17, which likely migrated from the Central Asia steppe (Sharma et al., 2009; Underhill et al., 2010). Unlike the profile of Y-chromosome haplogroups in Hui_Guizhou people, Han_Guizhou samples had high-frequency subclades of haplogroup O1a and O2a, which are the major local paternal lineages in East and Southeast Asia (van Oven et al., 2012; Yan et al., 2014). In particular, the haplogroups O1a1a2a1-CTS701, O1a1a1b1-Z23406, and O1a1a1a1a1a1a1a-A12439 in Han_Guizhou people were sublineages of O1a-M119, which was prevalent along the southeast coast of China (Wang and Li, 2013). The subhaplogroup of O2a2b1a1a-F8, F42 were suggested to be one of the three super-grandfathers for present-day Chinese that experienced star-like expansions in the Neolithic Era about 5.4 thousand years ago (Yan et al., 2014). Compared with Han Chinese in Guizhou, Hui_Guizhou people on the paternal perspective had more lineages that were frequent in North Asia and Central Asia.

## Discussion

The Hui people are a relatively small population (0.5% of the population of the province) but have a long history living in Guizhou province in southwest China. According to historical records, the Hui people came to Weining County in Guizhou province during the period of the early formation of Hui population in China (Bao, 1982; Yu, 2015). However, the origin and migration history of Hui people has always been obscure, due to the complicated ethnic origins and the lack of genome-wide data. Therefore, research on the Hui people in Guizhou is helpful for us to understand the early migration pattern and historical practice of the Hui people in China. In this study, we generated genome-wide SNP data from Hui and local Han Chinese people in Guizhou province. We merged our data with previously published datasets involving ancient and present-day populations and comprehensively integrated the genetic evidence of maternal, paternal and autosomal results to infer the origin and admixture history of the Hui people in Guizhou.

### Male-Driven Migration of Guizhou Hui

From the paternal perspective, we found the Y-chromosome lineages that are frequent in North Asia and Central Asia (Yan et al., 2014; Ilumäe et al., 2016) reached a higher frequency in Guizhou Hui people than in Guizhou Han Chinese. However, the paternal profile of Guizhou Han was quite different from Guizhou Hui by mainly having lineages that are predominant in East and Southeast Asia. However, on the maternal side, we found Guizhou Hui and Han shared almost all the mtDNA haplogroups. The inconsistency of paternal and maternal genetic profile indicated the migration practice of Guizhou Hui people was probably a sex-biased male-driven process. We noted the small number of male individuals in Guizhou Hui samples was a limitation of the study. However, the observed sex-biased pattern in Hui people were consistent with previous studies (Wang et al., 2019; Xie et al., 2019), as well as historical records (Bao, 1982; Mu, 1985; Xie and Shan, 2002) that the ancestors of Hui people migrated to China were mainly men. Their marriage was carried out in a relatively closed system of endogamy and the intermarriage usually involved indigenous Han women converting to Islam Hui when married to the Hui males (Gladney, 1998).

### Genetic Assimilation With Indigenous Han People

We found genetic evidence in Hui to support their intense admixture with indigenous Han people. The maternal lineages had shown Guizhou Han shared the majority of mtDNA haplogroups with Guizhou Hui people, such as B5, D4, C7, B4, A17, A14, and F. In addition, the *qpAdm*-based admixture models also demonstrated that Guizhou Hui shared more alleles with Guizhou Han than with other East Asian populations. The Guizhou Han related ancestry ranged from 70 to 85% in Guizhou Hui samples. These results indicate there have been massive assimilations of indigenous Han populations in the formation of Guizhou Hui people. The genetic affinity between Guizhou Hui and Guizhou Han is consistent with the previous evidence that the genetic profile of Muslim Hui people was closest to the indigenous Han Chinese populations (Xie and Shan, 2002; Zhang et al., 2010; Lan et al., 2018; Xie et al., 2019). Although the culture of Hui people has distinct differences with Han Chinese due to their practice of Islam, the majority of Hui people speak Han Chinese languages nowadays showing the close affinity with Han Chinese.

### Limited West Eurasian Related Ancestry in Guizhou Hui People

On the genome-wide side, in addition to the predominant East Asian related ancestry, we also detected there was Western Eurasian related ancestry in Guizhou Hui people. The results of *qpADM*-based admixture models indicated that Guizhou Hui individuals were estimated to have about 6% French related West Eurasian ancestry. We here used French as a proxy to estimate the admixture proportion, but we note the West Eurasian ancestry might be diverse and probably from different regions of West Eurasia. The origin and diversification of Hui groups in China via demic diffusion involving the mass movement of people or simple cultural diffusion is a long-going debate. Here we show the proportion of West Eurasian related ancestry in quite low in present-day Hui people, supporting the formation of Hui involving massive assimilation of indigenous East Asians.

### The North to South Migration of Guizhou Hui People

According to the historical records, since Yuan, Ming and Qing dynasties, Muslim Hui people had spread from Central Asia, Arabia, and Persia to China along the Silk Road Economic Belt (Mu, 1985; Xie and Shan, 2002; Yao et al., 2004). However, it was obscure from where and how the ancestors of Guizhou Hui came to southwest China. Our genetic evidence indicated the Guizhou Hui people showed an affinity with northern populations, such as Tibetan and Tungusic/Mongolic-speaking populations. We found the Guizhou Hui samples could be well modeled as a three-way admixture between Guizhou Han (69.6%), Tibetan (24%), and French (6.4%), or as a mixture of Guizhou Han (77.1%), Daur (16.3%), and French (6.6%). This was not caused by the recent expansion and gene flow of Han Chinese since we can get a similar admixture proportion when using the Neolithic DevilsCave samples as a source in place of present-day Tibetan/Daur. We have also found evidence to support this northern ancestry from paternal Y-chromosome of Guizhou Hui people. We proposed that the Muslim Hui people probably migrated into China via the northern route, then migrated southward into Guizhou province with the massive admixture with the indigenous ethnic groups, especially Han Chinese people, to form the present-day Guizhou Hui group.

In the future work, a larger number of samples from diverse Hui people living in different regions of China were considered to comprehensively reveal the genetic diversity and population history of the Hui ethnic groups.

## Conclusion

The Guizhou Hui group has a long history and unique cultural features. However, the origin and migration history of Hui people have always been obscure due to the lack of genome-wide data. Research on the Guizhou Hui people will help to provide clues to the long debate on the origin and diversification of the Muslim Hui people in China. In this study, from a genome-wide perspective, we synthetically integrated the genetic results of maternal mtDNA, paternal Y-chromosome and autosomal DNA. We proposed that the present-day Guizhou Hui people were formed by male-dominated migration of West Eurasian related people to East Asia with massive assimilation of indigenous East Asians, especially Han Chinese.

## Data Availability Statement

The data presented in the study are deposited in the zenodo repository, accession number (doi: 10.5281/zenodo.4275668).

## Ethics Statement

The studies involving human participants were reviewed and approved by the Medical Ethics Committee of Guizhou Medical University and Xiamen University (Approval Number: XDYX2019009) and were in accordance with the recommendations provided by the revised Helsinki Declaration of 2000. The patients/participants provided their written informed consent to participate in this study.

## Author Contributions

JH and C-CW designed this study. JZ and C-CW wrote the manuscript. QW, ZR, HZ, JJ, YLiu, MY, and JH collected the samples. QW, ZR, HZ, JJ, YLiu, MY, YLi, GC, and JH conducted the experiment. JZ, JS, GH, JG, XY, JC, KZ, RW, and C-CW analyzed the data. All authors reviewed the manuscript.

## Conflict of Interest

GC was employed by company WeGene. The remaining authors declare that the research was conducted in the absence of any commercial or financial relationships that could be construed as a potential conflict of interest. The handling editor declared a past co-authorship with one of the authors C-CW.

## Footnotes

1 https://reich.hms.harvard.edu/downloadable-genotypes-present-day-and-ancient-dna-data-compiled-published-papers

2 http://www.isogg.org/tree/

3 http://www.phylotree.org/

## Supplementary Material

Supplementary Figure 1 Heatmap showed the shared genetic drift of Hui_Guizhou and Han_Guizhou with other present and ancient populations, measured by Outgroup *f*3 statistics (Source1, Source2; Mbuti). The Hui_Guizhou and Han_Guizhou groups marked by the red and blue wireframe, respectively. Lighter colors indicate more shared drift.

Supplementary Figure 2 Results of admixture *f3*-statistics of the form *f3* (X, Target; Mbuti). We only plotted the *f3*-values of top 50 populations related to Hui_Guizhou people.

Supplementary Figure 3 Admixture analysis with 56 East Asian and Western related populations assuming 2–10 ancestral populations. Our sample groups were marked by red wireframe.
