# Supplementary figures and images for: Male-Dominated Migration and Massive Assimilation of Indigenous East Asians in the Formation of Muslim Hui People in Southwest China
Source: Front Genet. 2021 Jan 11;11:618614. doi: 10.3389/fgene.2020.618614 (PMC7834311; doi:10.3389/fgene.2020.618614)

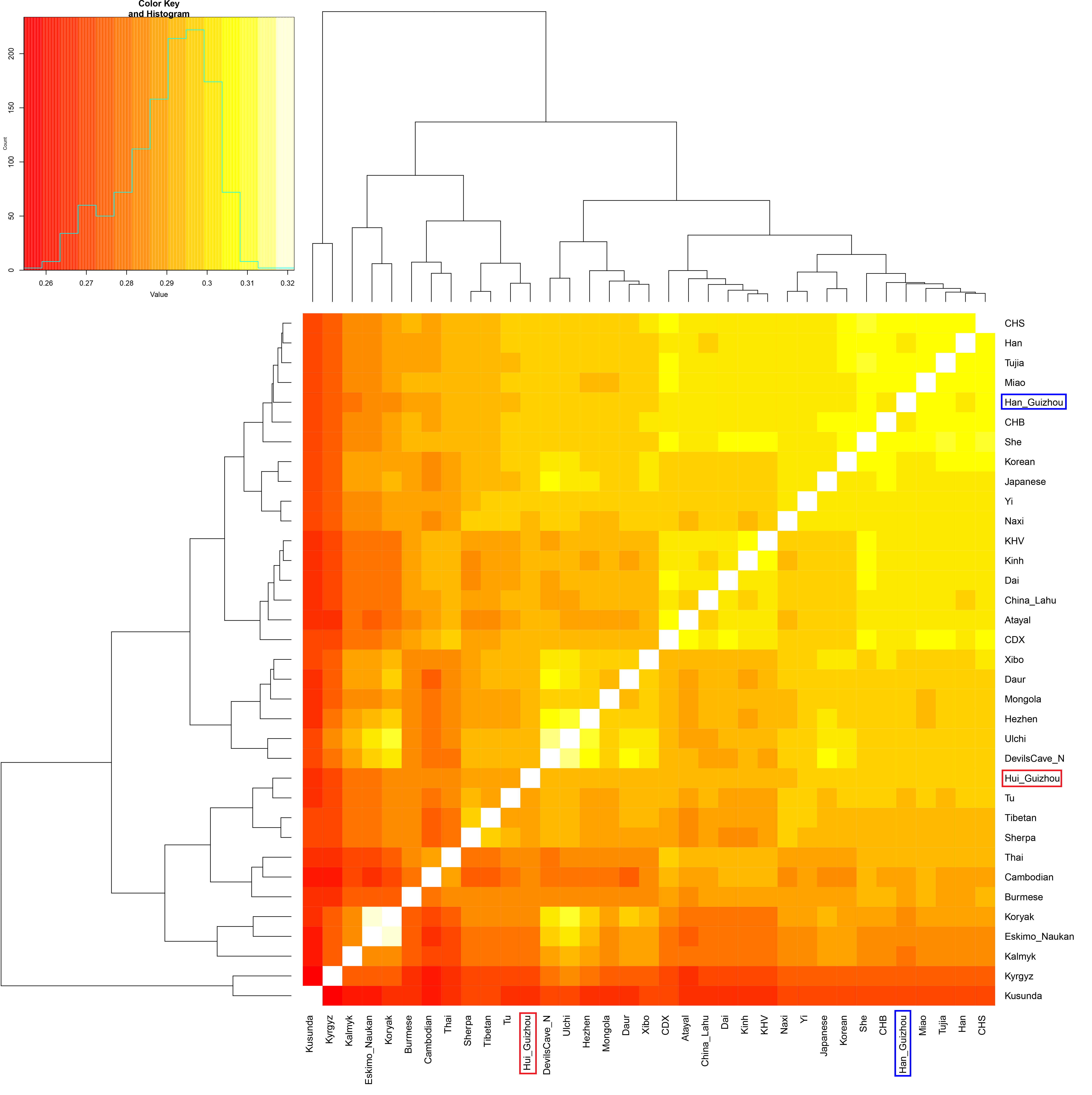

Supplement: Supplementary Figure 1 — Heatmap showed the shared genetic drift of Hui_Guizhou and Han_Guizhou with other present and ancient populations, measured by Outgroup f3 statistics (Source1, Source2; Mbuti). The Hui_Guizhou and Han_Guizhou groups marked by the red and blue wireframe, respectively. Lighter colors indicate more shared drift. [file Image_1.JPEG]

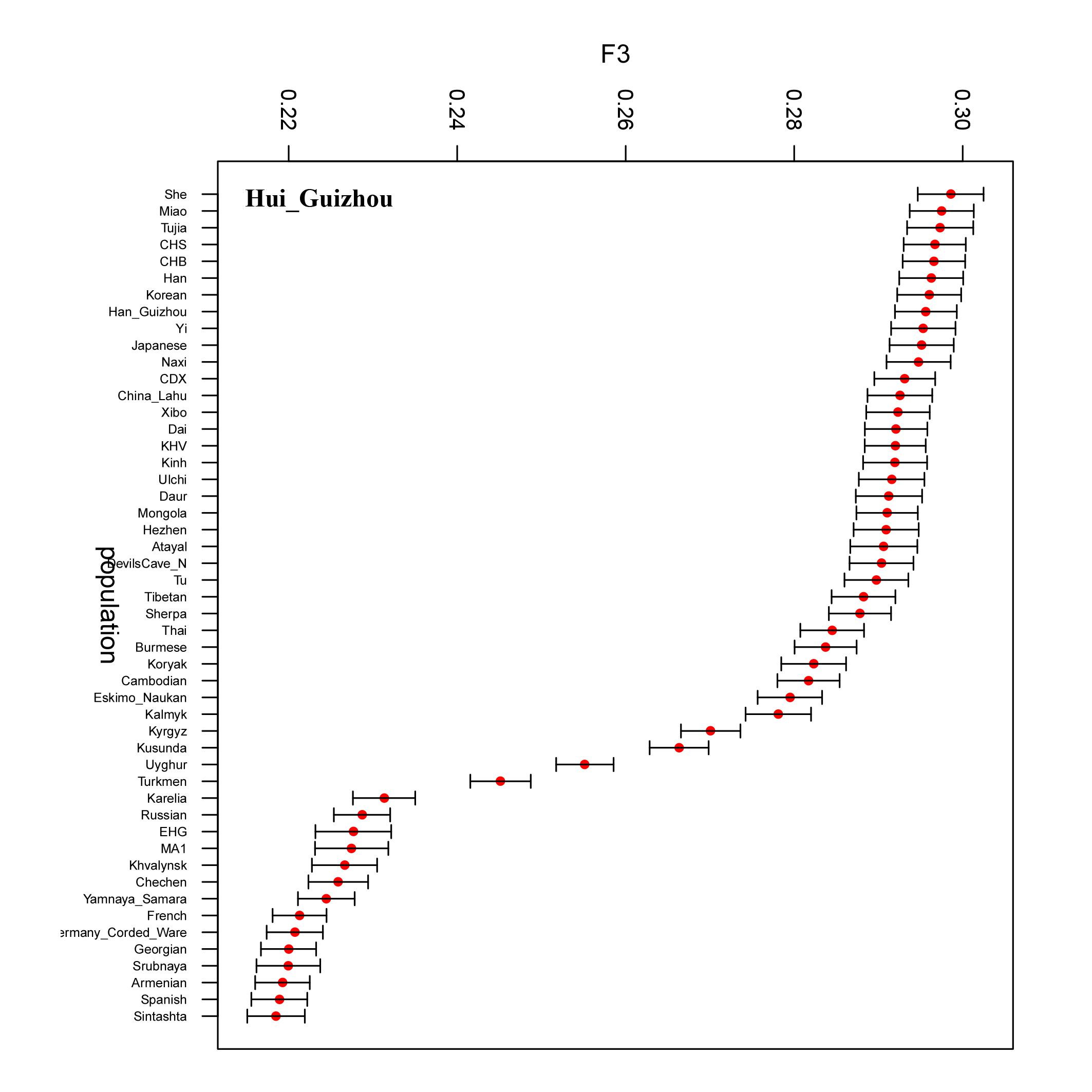

Supplement: Supplementary Figure 2 — Results of admixture f3-statistics of the form f3 (X, Target; Mbuti). We only plotted the f3-values of top 50 populations related to Hui_Guizhou people. [file Image_2.JPEG]
