# Supplementary material for: Male-Dominated Migration and Massive Assimilation of Indigenous East Asians in the Formation of Muslim Hui People in Southwest China
Source: Front Genet. 2021 Jan 11;11:618614. doi: 10.3389/fgene.2020.618614 (PMC7834311; doi:10.3389/fgene.2020.618614)
Supplement: Supplementary file 4 [file Table_1.DOCX]

**Supplemental materials**

**Table S1** Summary of context for the 45 Hui amd 14 Han individuals in Guizhou province

| **ID** | **Sex** | **Group** | **Location** | **Province** |
| --- | --- | --- | --- | --- |
| 10091808171506 | Male | Hui | Gali Village in Mazha Town, Weining County | Guizhou |
| 11601808171276 | Male | Hui | Gali Village in Mazha Town, Weining County | Guizhou |
| 14591808171232 | Female | Hui | Gali Village in Mazha Town, Weining County | Guizhou |
| 15001702300008 | Male | Hui | Gali Village in Mazha Town, Weining County | Guizhou |
| 15001702300023 | Male | Hui | Gali Village in Mazha Town, Weining County | Guizhou |
| 15001702300028 | Female | Hui | Gali Village in Mazha Town, Weining County | Guizhou |
| 15001702300045 | Male | Hui | Gali Village in Mazha Town, Weining County | Guizhou |
| 15001702300077 | Female | Hui | Gali Village in Mazha Town, Weining County | Guizhou |
| 15001702300082 | Female | Hui | Gali Village in Mazha Town, Weining County | Guizhou |
| 15001702300084 | Female | Hui | Gali Village in Mazha Town, Weining County | Guizhou |
| 15001702300112 | Female | Hui | Gali Village in Mazha Town, Weining County | Guizhou |
| 15001702300138 | Female | Hui | Gali Village in Mazha Town, Weining County | Guizhou |
| 15001702300173 | Female | Hui | Gali Village in Mazha Town, Weining County | Guizhou |
| 15001702300176 | Male | Hui | Gali Village in Mazha Town, Weining County | Guizhou |
| 15001702300202 | Female | Hui | Gali Village in Mazha Town, Weining County | Guizhou |
| 15001702300203 | Male | Hui | Gali Village in Mazha Town, Weining County | Guizhou |
| 15001702300205 | Female | Hui | Gali Village in Mazha Town, Weining County | Guizhou |
| 15001702300206 | Male | Hui | Gali Village in Mazha Town, Weining County | Guizhou |
| 15001702300209 | Female | Hui | Gali Village in Mazha Town, Weining County | Guizhou |
| 15001702300217 | Male | Hui | Gali Village in Mazha Town, Weining County | Guizhou |
| 15001702300230 | Male | Hui | Gali Village in Mazha Town, Weining County | Guizhou |
| 16341808209803 | Female | Hui | Gali Village in Mazha Town, Weining County | Guizhou |
| 19181808171501 | Female | Hui | Gali Village in Mazha Town, Weining County | Guizhou |
| 19721808171477 | Male | Hui | Gali Village in Mazha Town, Weining County | Guizhou |
| 24941808171400 | Female | Hui | Gali Village in Mazha Town, Weining County | Guizhou |
| 27301808171395 | Male | Hui | Gali Village in Mazha Town, Weining County | Guizhou |
| 28831808191334 | Male | Hui | Gali Village in Mazha Town, Weining County | Guizhou |
| 30291808191346 | Female | Hui | Gali Village in Mazha Town, Weining County | Guizhou |
| 30951808171514 | Female | Hui | Gali Village in Mazha Town, Weining County | Guizhou |
| 35981808171434 | Male | Hui | Gali Village in Mazha Town, Weining County | Guizhou |
| 37141808171482 | Female | Hui | Gali Village in Mazha Town, Weining County | Guizhou |
| 41111808174639 | Male | Hui | Gali Village in Mazha Town, Weining County | Guizhou |
| 41141808209765 | Female | Hui | Gali Village in Mazha Town, Weining County | Guizhou |
| 43581808174601 | Female | Hui | Gali Village in Mazha Town, Weining County | Guizhou |
| 44121808191322 | Female | Hui | Gali Village in Mazha Town, Weining County | Guizhou |
| 46251808171470 | Female | Hui | Gali Village in Mazha Town, Weining County | Guizhou |
| 51931808209774 | Female | Hui | Gali Village in Mazha Town, Weining County | Guizhou |
| 55081808209759 | Female | Hui | Gali Village in Mazha Town, Weining County | Guizhou |
| 60401808171499 | Female | Hui | Gali Village in Mazha Town, Weining County | Guizhou |
| 60631808171422 | Female | Hui | Gali Village in Mazha Town, Weining County | Guizhou |
| 64931808191345 | Female | Hui | Gali Village in Mazha Town, Weining County | Guizhou |
| 66801808209822 | Female | Hui | Gali Village in Mazha Town, Weining County | Guizhou |
| 67241808191313 | Female | Hui | Gali Village in Mazha Town, Weining County | Guizhou |
| 69701808171455 | Female | Hui | Gali Village in Mazha Town, Weining County | Guizhou |
| 73001808171427 | Female | Hui | Gali Village in Mazha Town, Weining County | Guizhou |
| 25461808178912 | Male | Han | Guiyang City | Guizhou |
| 32481808175474 | Female | Han | Guiyang City | Guizhou |
| 32731808175485 | Male | Han | Guiyang City | Guizhou |
| 34671808175734 | Female | Han | Guiyang City | Guizhou |
| 39771808186953 | Male | Han | Guiyang City | Guizhou |
| 40321808178750 | Male | Han | Guiyang City | Guizhou |
| 42311808189234 | Female | Han | Guiyang City | Guizhou |
| 45551808189658 | Male | Han | Guiyang City | Guizhou |
| 58781808175701 | Male | Han | Guiyang City | Guizhou |
| 64351808176770 | Female | Han | Guiyang City | Guizhou |
| 64451808176615 | Female | Han | Guiyang City | Guizhou |
| 69791808179035 | Male | Han | Guiyang City | Guizhou |
| 73321808176737 | Male | Han | Guiyang City | Guizhou |
| 73661808176785 | Female | Han | Guiyang City | Guizhou |

**Table S2** Statistics of the form of *f*3(Target; Source1, Source2), where Source1 and Source2 are worldwide populations. Here we only list the top 15 negative *f*3 values for each Target population.

| **Source 1** | **Source 2** | **Target** | ***f_3_*** | **std.err** | **Z** |
| --- | --- | --- | --- | --- | --- |
| Atayal | Tibetan | Han_Guizhou | -0.00536 | 0.001358 | -3.945 |
| Atayal | Ulchi | Han_Guizhou | -0.003305 | 0.001395 | -2.368 |
| Atayal | Hezhen | Han_Guizhou | -0.003287 | 0.00129 | -2.548 |
| Atayal | Eskimo_Naukan | Han_Guizhou | -0.003208 | 0.001511 | -2.123 |
| Dai | Tibetan | Han_Guizhou | -0.00286 | 0.000637 | -4.486 |
| Cambodian | Daur | Han_Guizhou | -0.002616 | 0.001178 | -2.221 |
| Atayal | Romania_C | Han_Guizhou | -0.002599 | 0.002054 | -1.265 |
| Kalmyk | She | Han_Guizhou | -0.002579 | 0.001172 | -2.2 |
| Atayal | Korean | Han_Guizhou | -0.002566 | 0.001195 | -2.147 |
| Atayal | Xibo | Han_Guizhou | -0.002489 | 0.001252 | -1.988 |
| CDX | Tibetan | Han_Guizhou | -0.00243 | 0.000444 | -5.475 |
| Dai | Koryak | Han_Guizhou | -0.002241 | 0.000872 | -2.569 |
| Dai | Sherpa | Han_Guizhou | -0.002175 | 0.000688 | -3.164 |
| Atayal | Kyrgyz | Han_Guizhou | -0.002174 | 0.001117 | -1.946 |
| Atayal | Naxi | Han_Guizhou | -0.002174 | 0.001039 | -2.091 |
| She | Spanish | Hui_Guizhou | -0.002707 | 0.001068 | -2.535 |
| Russia_Khvalynsk | She | Hui_Guizhou | -0.002232 | 0.001462 | -1.527 |
| Han_Guizhou | Amphora | Hui_Guizhou | -0.002064 | 0.000687 | -3.005 |
| Han | Amphora | Hui_Guizhou | -0.001966 | 0.001034 | -1.902 |
| Amphora | She | Hui_Guizhou | -0.001688 | 0.001338 | -1.261 |
| Han_Guizhou | Hungary | Hui_Guizhou | -0.001565 | 0.000669 | -2.338 |
| Han_Guizhou | Spanish | Hui_Guizhou | -0.001558 | 0.00055 | -2.834 |
| CHS | Sintashta | Hui_Guizhou | -0.001524 | 0.000529 | -2.882 |
| French | She.DG | Hui_Guizhou | -0.001474 | 0.000943 | -1.563 |
| CHB | Sintashta | Hui_Guizhou | -0.001416 | 0.000537 | -2.636 |
| Han_Guizhou | Yamnaya_Samara | Hui_Guizhou | -0.001398 | 0.000555 | -2.52 |
| Miao | Russia_Srubnaya | Hui_Guizhou | -0.001379 | 0.001356 | -1.017 |
| Atayal | Romania_C | Hui_Guizhou | -0.001341 | 0.002086 | -0.643 |
| Han_Guizhou | Armenian | Hui_Guizhou | -0.001227 | 0.000535 | -2.294 |
| Han_Guizhou | French | Hui_Guizhou | -0.001216 | 0.000475 | -2.562 |

**Table S3** *f*4-statistics in the form of *f4*(Test, Mbuti ; Hui_Guizhou, Han_Guizhou)

| **Test** | ***f4*(Test, Mbuti ; Hui_Guizhou, Han_Guizhou)** | |
| --- | --- | --- |
|  | *f_4_* | Zscore |
| Atayal | -0.0207 | -11.521 |
| She | -0.0177 | -11.272 |
| CDX | -0.0175 | -13.98 |
| Dai | -0.0173 | -12.741 |
| CHS | -0.0161 | -12.555 |
| KHV | -0.0161 | -12.918 |
| Han | -0.0159 | -11.004 |
| Tujia | -0.0152 | -9.954 |
| Korean | -0.0149 | -9.909 |
| Cambodian | -0.0147 | -9.897 |
| CHB | -0.0146 | -11.539 |
| Miao | -0.0146 | -9.208 |
| Yi | -0.0133 | -8.532 |
| Xibo | -0.0124 | -8.442 |
| Naxi | -0.0122 | -8.261 |
| Hezhen | -0.0115 | -7.527 |
| Ulchi | -0.0105 | -6.832 |
| Daur | -0.0103 | -5.714 |
| DevilsCave_N | -0.0101 | -6.259 |
| Tibetan | -0.0094 | -6.323 |
| Romania_C | 0.0061 | 3.428 |
| Sardinian | 0.0068 | 5.006 |
| French | 0.0082 | 6.174 |
| Yamnaya_Samara | 0.0087 | 6.172 |
| Amphora | 0.0091 | 4.932 |

**Table S4** *f*4-statistics in the form of *f4*(Test, Mbuti ; Hui_Guizhou, Han)

| **Test** | ***f4*(Test, Mbuti ; Hui_Guizhou, Han)** | |
| --- | --- | --- |
|  | *f_4_* | Zscore |
| Spanish | 0.0072 | 3.191 |
| French | 0.006 | 2.717 |
| Amphora | 0.008 | 2.712 |
| Anatolia_N | 0.0054 | 2.606 |
| Georgian | 0.0054 | 2.363 |
| Atayal | -0.0237 | -8.372 |
| CDX | -0.0209 | -10.472 |
| CHS | -0.0194 | -9.753 |
| She | -0.0191 | -7.91 |
| Dai | -0.0189 | -8.772 |
| Korean | -0.0181 | -7.937 |
| Tujia | -0.018 | -7.717 |
| CHB | -0.0178 | -8.989 |
| Japanese | -0.0171 | -7.447 |
| Thai | -0.0171 | -7.489 |
| Mlabri | -0.0167 | -6.676 |
| Miao | -0.0164 | -7.276 |
| Htin_Mal | -0.0155 | -7.157 |
| Naxi | -0.0152 | -6.93 |
| Yi | -0.0142 | -6.021 |
| Tu | -0.014 | -6.158 |
| Burmese | -0.0139 | -6.21 |
| Hezhen | -0.0138 | -6.013 |
| China_Lahu | -0.0136 | -5.634 |
| Sherpa | -0.0135 | -5.614 |
| Daur | -0.0126 | -4.779 |
| Mongola | -0.0123 | -5.429 |
| Xibo | -0.0122 | -5.477 |

**Table S5** *f*4-statistics in the form of *f4*(Test, Mbuti ; Hui_Guizhou, She)

| **Test** | ***f4*(Test, Mbuti ; Hui_Guizhou, She)** | |
| --- | --- | --- |
|  | *f_4_* | Zscore |
| Spanish | 0.009 | 2.965 |
| French | 0.0059 | 2.138 |
| Khvalynsk_EN | 0.0095 | 2.328 |
| Anatolia_N | 0.005 | 1.787 |
| Atayal | -0.0279 | -7.601 |
| Dai | -0.0256 | -9.13 |
| CDX | -0.0251 | -9.936 |
| CHS | -0.0232 | -9.184 |
| Tujia | -0.023 | -7.351 |
| Mlabri | -0.0219 | -7.049 |
| Htin_Mal | -0.0216 | -7.785 |
| Han | -0.0211 | -7.482 |
| CHB | -0.0201 | -7.986 |
| China_Lahu | -0.02 | -6.7 |
| Thai | -0.0199 | -6.492 |
| Burmese | -0.0183 | -5.853 |
| Japanese | -0.0177 | -5.883 |
| Yi | -0.0172 | -5.387 |
| Daur | -0.0167 | -4.757 |
| Hezhen | -0.0164 | -5.205 |
| Naxi | -0.0156 | -5.158 |
| Ulchi | -0.0148 | -4.688 |
| Xibo | -0.0137 | -4.427 |
| Mongola | -0.0136 | -4.403 |
| Tu | -0.0135 | -4.231 |
| Eskimo_Naukan | -0.0119 | -3.512 |
| Tibetan.DG | -0.0106 | -3.323 |

**Table S6** *f*4-statistics in the form of *f4*(Test, Mbuti ; Hui_Guizhou, Miao)

| **Test** | ***f4*(Test, Mbuti ; Hui_Guizhou, Miao)** | |
| --- | --- | --- |
|  | *f_4_* | Zscore |
| French | 0.0074 | 2.656 |
| Srubnaya | 0.0085 | 2.23 |
| Khvalynsk_EN | 0.0071 | 1.677 |
| Anatolia_N | 0.0043 | 1.532 |
| Htin_Mal | -0.0222 | -7.784 |
| CDX | -0.0211 | -7.978 |
| China_Lahu | -0.0208 | -6.456 |
| Dai | -0.02 | -7.123 |
| Mlabri | -0.0197 | -5.911 |
| Atayal | -0.0196 | -5.196 |
| She | -0.0191 | -5.852 |
| Tujia | -0.0181 | -5.625 |
| Thai | -0.0177 | -6.082 |
| CHS | -0.0171 | -6.477 |
| Han | -0.0161 | -5.746 |
| Burmese | -0.0152 | -4.864 |
| CHB | -0.0151 | -5.71 |
| Naxi | -0.0138 | -4.813 |
| Korean | -0.0136 | -4.331 |
| Japanese | -0.0133 | -4.405 |
| Tu | -0.0129 | -4.127 |
| Yi | -0.0129 | -3.977 |
| Ulchi | -0.0127 | -3.852 |
| Tibetan | -0.0101 | -3.21 |

**Table S7** Hui_Guizhou people-related admixture proportions estimated using qpAdm. Here “p” refers to the P-value for rank=2 and “std.err” is the standard error estimated using jackknife. We used qpAdm to estimate Hui_Guizhou people ancestry using Mbuti, Ust_Ishim, ONG, Papuan, Russia_Kostenki14, Australian, Surui, Eskimo_Naukan, Mixe and Tibetan as outgroups. Han_Guizhou and French were as proxies for the source populations.

| **Test** | ***p* Value** | **Han_Guizhou** | | **French** | |
| --- | --- | --- | --- | --- | --- |
|  | rank=2 | proportion | std.err | proportion | std.err |
| **Hui_Guizhou** | 0.20201 | 0.938 | 0.007 | 0.062 | 0.007 |

**Table S8** We replaced Han_Guizhou with Chinese Han in datasets to repeatedly estimate Hui_Guizhou people-related admixture proportions using qpAdm. Here “p” refers to the P-value for rank=2 and “std.err” is the standard error estimated using jackknife. Mbuti, Ust_Ishim, ONG, Papuan, Russia_Kostenki14, Australian, Surui, Eskimo_Naukan, Mixe and Tibetan were as outgroups.

| **Test** | ***p* Value** | **Han** | | **French** | |
| --- | --- | --- | --- | --- | --- |
|  | rank=2 | proportion | std.err | proportion | std.err |
| **Hui_Guizhou** | 0.43948 | 0.937 | 0.011 | 0.063 | 0.011 |

**Table S9** Hui_Guizhou people-related admixture proportions estimated using qpAdm. Here “p” refers to the P-value for rank=2 and “std.err” is the standard error estimated using jackknife. We used qpAdm to estimate Hui_Guizhou people ancestry using Mbuti, Ust_Ishim, ONG, Papuan, Australian, Surui, Russia_MA1_HG, Romania_Oase1, Russia_Yamnaya_Samara, Georgian, Germany_Corded_Ware, Hungary_LCA, Kusunda, Spanish and Atayal as outgroups. Han_Guizhou, Tibetan and French were as proxies for the source populations.

| **Test** | ***p* Value** | **Han_Guizhou** | | **Tibetan** | | **French** | |
| --- | --- | --- | --- | --- | --- | --- | --- |
|  | rank=2 | proportion | std.err | proportion | std.err | proportion | std.err |
| **Hui_Guizhou** | 0.97991 | 0.696 | 0.038 | 0.240 | 0.041 | 0.064 | 0.005 |

**Table S10** Hui_Guizhou people-related admixture proportions estimated using qpAdm. Here “p” refers to the P-value for rank=2 and “std.err” is the standard error estimated using jackknife. We used qpAdm to estimate Hui_Guizhou people ancestry using Mbuti, Ust_Ishim, ONG, Russia_Kostenki14, Papuan, Australian, Mixe, Surui, Eskimo_Naukan, Russia_MA1_HG, Romania_Oase1, Atayal and Russia_Yamnaya_Samara as outgroups. Han_Guizhou, DevilsCave and French were as proxies for the source populations.

| **Test** | ***p* Value** | **Han_Guizhou** | | **DevilsCave** | | **French** | |
| --- | --- | --- | --- | --- | --- | --- | --- |
|  | rank=2 | proportion | std.err | proportion | std.err | proportion | std.err |
| **Hui_Guizhou** | 0.11712 | 0.736 | 0.048 | 0.174 | 0.048 | 0.090 | 0.005 |

**Table S11** Hui_Guizhou people-related admixture proportions estimated using qpAdm. Here “p” refers to the P-value for rank=2 and “std.err” is the standard error estimated using jackknife. We used qpAdm to estimate Hui_Guizhou people ancestry using Mbuti, Ust_Ishim, ONG, Papuan, Russia_Kostenki14, Australian, Surui, Eskimo_Naukan, Mixe, Russia_MA1_HG, Romania_Oase1, Georgian and Russia_Yamnaya_Samara as outgroups. Han_Guizhou, Ulchi and French were as proxies for the source populations.

| **Test** | ***p* Value** | **Han_Guizhou** | | **Ulchi** | | **French** | |
| --- | --- | --- | --- | --- | --- | --- | --- |
|  | rank=2 | proportion | std.err | proportion | std.err | proportion | std.err |
| **Hui_Guizhou** | 0.40926 | 0.850 | 0.038 | 0.074 | 0.036 | 0.076 | 0.005 |

**Table S12** Hui_Guizhou people-related admixture proportions estimated using qpAdm. Here “p” refers to the P-value for rank=2 and “std.err” is the standard error estimated using jackknife. We used qpAdm to estimate Hui_Guizhou people ancestry using Mbuti, Ust_Ishim, ONG, Papuan, Russia_Kostenki14, Australian, Surui, Eskimo_Naukan, Mixe, Russia_MA1_HG, Romania_Oase1, Georgian and Russia_Yamnaya_Samara as outgroups. Han_Guizhou, Daur and French were as proxies for the source populations.

| **Test** | ***p* Value** | **Han_Guizhou** | | **Daur** | | **French** | |
| --- | --- | --- | --- | --- | --- | --- | --- |
|  | rank=2 | proportion | std.err | proportion | std.err | proportion | std.err |
| **Hui_Guizhou** | 0.62959 | 0.771 | 0.075 | 0.163 | 0.078 | 0.066 | 0.006 |

**Table S13** Paternal Y chromosome DNA and maternal mtDNA haplogroups assignment

| **ID** | **Group** | **mtDNA** | **Y chromosome** |
| --- | --- | --- | --- |
| 10091808171506 | Hui_Guizhou | A17 | Q1b2b1b2a-L330-F1893 |
| 11601808171276 | Hui_Guizhou | D6c | Q1b2b1b2a-L330-F1893 |
| 14591808171232 | Hui_Guizhou | D4 | NA |
| 15001702300008 | Hui_Guizhou | C7a1c | C2b1a2b2- FGC45548 |
| 15001702300023 | Hui_Guizhou | A5b1b | R1a1a1b2-M17-F992 |
| 15001702300028 | Hui_Guizhou | M8a3 | NA |
| 15001702300045 | Hui_Guizhou | G2a2a | Q1b2b1b2a-L330-F1893 |
| 15001702300077 | Hui_Guizhou | B4g | NA |
| 15001702300082 | Hui_Guizhou | B5b2a1 | NA |
| 15001702300084 | Hui_Guizhou | F2a | NA |
| 15001702300112 | Hui_Guizhou | D5b1d | NA |
| 15001702300138 | Hui_Guizhou | B4g | NA |
| 15001702300173 | Hui_Guizhou | B4b1a1 | NA |
| 15001702300176 | Hui_Guizhou | F2 | Q1b2b1b2a-L330-F1893 |
| 15001702300202 | Hui_Guizhou | B4g | NA |
| 15001702300203 | Hui_Guizhou | D4g2a1 | Q1b2b1b2a-L330-F1893 |
| 15001702300205 | Hui_Guizhou | D4j | NA |
| 15001702300206 | Hui_Guizhou | B4g | Q1b2b1b2a-L330-F1893 |
| 15001702300209 | Hui_Guizhou | D4 | NA |
| 15001702300217 | Hui_Guizhou | D4 | N1b2a2-F2930-M1811 |
| 15001702300230 | Hui_Guizhou | D4j | R1a1a1b1a2b3-M17-FGC4499 |
| 16341808209803 | Hui_Guizhou | B4g | NA |
| 19181808171501 | Hui_Guizhou | D4g2a1 | NA |
| 19721808171477 | Hui_Guizhou | D4j | Q1b2b1b2a-L330-F1893 |
| 24941808171400 | Hui_Guizhou | A17 | NA |
| 27301808171395 | Hui_Guizhou | B6a | N1b2a2-F2930-M1811 |
| 28831808191334 | Hui_Guizhou | D4j | N1b2a2-F2930-M1811 |
| 30291808191346 | Hui_Guizhou | M11a2 | NA |
| 30951808171514 | Hui_Guizhou | D4a5 | NA |
| 35981808171434 | Hui_Guizhou | M11a2 | Q1b2b1b2a-L330-F1893 |
| 37141808171482 | Hui_Guizhou | C4a1d | NA |
| 41111808174639 | Hui_Guizhou | A17 | N1b2a2-F2930-M1811 |
| 41141808209765 | Hui_Guizhou | M8a3a | NA |
| 43581808174601 | Hui_Guizhou | D4b1a | NA |
| 44121808191322 | Hui_Guizhou | C7a1c | NA |
| 46251808171470 | Hui_Guizhou | N9a1 | NA |
| 51931808209774 | Hui_Guizhou | A5b1b | NA |
| 55081808209759 | Hui_Guizhou | C7a | NA |
| 60401808171499 | Hui_Guizhou | C4 | NA |
| 60631808171422 | Hui_Guizhou | A14 | NA |
| 64931808191345 | Hui_Guizhou | R9b2 | NA |
| 66801808209822 | Hui_Guizhou | R9b1a3 | NA |
| 67241808191313 | Hui_Guizhou | N9a9 | NA |
| 69701808171455 | Hui_Guizhou | C7a1c | NA |
| 73001808171427 | Hui_Guizhou | D4g2a1 | NA |
| 25461808178912 | Han_Guizhou | F1a1 | O1a1a2a1-M119-CTS701 |
| 32481808175474 | Han_Guizhou | B5b1 | NA |
| 32731808175485 | Han_Guizhou | D4b2b | O1a1a1b1- M119-Z23406 |
| 34671808175734 | Han_Guizhou | C7a1 | NA |
| 39771808186953 | Han_Guizhou | B5b2a | O2a2b1a1a1a1a1-M122-Y17728 |
| 40321808178750 | Han_Guizhou | F3a1 | C2b1b-F845 |
| 42311808189234 | Han_Guizhou | B4b1a2a | NA |
| 45551808189658 | Han_Guizhou | D4a | N1b2a2-M1811 |
| 58781808175701 | Han_Guizhou | A | O2a2b1a1a-M122-M133 |
| 64351808176770 | Han_Guizhou | A | NA |
| 64451808176615 | Han_Guizhou | Z3 | NA |
| 69791808179035 | Han_Guizhou | B5a | O1a1a1a1a1a1a1a-M119-A12439 |
| 73321808176737 | Han_Guizhou | A14 | O2a2b1a2a1a3b-M122-F3386 |
| 73661808176785 | Han_Guizhou | A17 | NA |
